# Investigation of the Possibility of Using Microspectrometers Based on CMOS Photodiode Arrays in Small-Sized Devices for Optical Diagnostics

**DOI:** 10.3390/s22114195

**Published:** 2022-05-31

**Authors:** Oleksandra Hotra, Vladimir Firago, Nikolay Levkovich, Konstantin Shuliko

**Affiliations:** 1 Department of Electronics and Information Technology, Lublin University of Technology, Nadbystrzycka 38D, 20-618 Lublin, Poland; 2 Department of Radiophysics and Computer Technologies, Belarusian State University, Nezavisimosti Avenue 4, 220030 Minsk, Belarus; firago@bsu.by (V.F.); leukovich@gmail.com (N.L.); konstantin.shuliko@gmail.com (K.S.)

**Keywords:** microspectrometers based on photodiode arrays, optical diagnostics, digital methods for spectra processing, spectrometer parameters, spectral resolution of the spectrometer

## Abstract

The article considers the potential applicability of C12880MA and C11708MA Hamamatsu microspectrometers, which are characterized by an extremely compact design, occupying a small volume of several cubic centimeters, in portable spectrometric equipment with spatial resolution for monitoring the optical properties of condensed scattering media. The development of methods for determining the reduced scattering and absorption spectral coefficients of radiation from various scattering materials and products allows us to speak about the possibility of real-time control of the volume concentration of optically active components included in them, for example, fat and water in dairy products. For this, it is necessary to provide sufficiently accurate spectra of diffusely reflected broadband light radiation at different distances between the points of radiation entrance and registration. The aim of the manuscript is to assess the possibility of using the considered microspectrometers in compact devices for optical diagnostics and control of the optical properties of condensed scattering media. The features of the connection diagram of these microspectrometers and the necessary methods for correcting the initially obtained spectral dependencies of diffusive reflection, which will be of interest to developers of spectral diagnostic equipment, are considered in detail. The need to eliminate the influence of the inhomogeneity of dark counts of a CMOS photodiode array is shown. The hardware functions of the C12880MA and C11708MA Hamammatsu microspectrometers, as well as the AvaSpec 2048L fiber-optic spectrometer, were experimentally measured and compared. Methods for correcting the nonlinearity of their reading scales and light characteristics, as well as improving their equivalent spectral resolution using digital Wiener filtering, are described. It is shown that the equivalent spectral resolution of C12880MA and C11708MA microspectrometers can be improved by about 40% when recording smooth spectra, subject to the condition that the resulting side oscillations are small. It is pointed out that in order to reduce the level of side oscillations in the corrected spectra with improved resolution, it is necessary to ensure the smoothness of the original spectra and a good signal-to-noise ratio. A conclusion is made about the possibility of using the considered microspectrometers in portable spectrometric equipment with careful consideration of their characteristics, the features of their switching circuit, and the necessary software.

## 1. Introduction

Achievements in the development of operational methods for spectrometric control of the composition of various materials and substances allow their implementation in everyday practice [1,2,3,4,5]. New methods of optical diagnostics appear, based both on the theory of radiation transfer in scattering media [6,7,8,9,10,11,12,13,14] and on original methods for determining the necessary parameters, for example, temperature [15] or gas composition [16]. Modern technologies of digital processing of registered signals simplify the use of these methods in different industries, agriculture, medicine, etc. [17]. An example is the use of spectral techniques in the visible and near-infrared spectral ranges in assessing the properties of fruits and vegetables, which are discussed in detail in [3,4,18,19]. In [5,20], the problems of real-time spectral control of the volume concentration of the main components of raw milk (fat, protein, and lactose) are analyzed. The spectroscopic determination of the concentration of fat in a raw milk stream is complicated by its strong scattering of transmission radiation. It is necessary to use thin cuvettes with a thickness of 1 mm, which determines the small optical path and low sensitivity of the equipment designed to determine the spectral absorption coefficient μ*_a_*(λ) of milk. When using diffuse reflection spectroscopy with spatial resolution, the length of the average optical path that photons pass between emitting and receiving probes can be more than 1 cm, which increases the sensitivity of real-time control by an order of magnitude.

However, for their widespread implementation, the corresponding spectrometric equipment should be compact [17,21], easy to use [22,23,24], and inexpensive.

The Hamamatsu Corporation has mastered the serial production of the corresponding microspectrometers, for example, C12880MA and C11708MA, which provide measurement of radiation spectra in the ranges from 340 to 850 nm and from 640 to 1050 nm, respectively [25,26]. One of the competitive niches of their application is portable spectrometric equipment with spatial resolution for monitoring the optical properties of condensed scattering media [2,3,6,7,8,9,10,11,12]. Since diffuse reflectance spectroscopy with spatial resolution provides more information compared to that using a single distance ρ between the point of radiation input into the scattering medium and the point of radiation registration, it becomes possible to determine the reduced scattering μ′*_s_*(λ) and absorption μ*_a_*(λ) spectral coefficients of the controlled medium. In this case, one has to compose systems of nonlinear equations and solve ill-posed inverse problems. Therefore, it is necessary to provide sufficiently accurate spectra of diffusely reflected broadband light radiation at different distances ρ*_k_* between the points of radiation entrance and registration. Note that with the correct determination of the spectral absorption coefficient of radiation μ*_a_*(λ), it is possible to solve further inverse problems to determine the volume concentration *C_Vm_* of the main chromophores of a controlled scattering medium with known spectral absorption coefficients μ*_am_*(λ) [27]. The noted features of recording the diffuse reflection spectra at different distances ρ*_k_* require conducting of the calibration of microspectrometers [28] with the elimination of the nonlinearity of their light characteristics. It is also necessary to minimize the influence of baseline inhomogeneity and temperature drift of the dark current of the used photodetector array.

The issue of increasing the spectral resolution of these microspectrometers requires separate consideration. In technical documentation for spectrometers based on arrays of photodetectors, the definition of the resolution *r* is usually used, based on the determination of the spectral width of a monochromatic source at a level of 50% of its maximum. Resonance absorption bands of substances in condensed media are usually broadened, and when analyzing their spectra in the visible and near-infrared ranges of electromagnetic radiation, a resolution of about 5 nm is often sufficient. Due to the short path of the beams in the C12880MA and C11708MA microspectrometers, their spectral resolution is worse, which can cause some smoothing of the peaks of the resonance absorption bands. When solving inverse problems for determining the volume concentration of concentrations of main chromophores *C_Vm_*, this can lead to distortion of the measurement results.

The purpose of this article is to experimentally evaluate the possibility of using the considered microspectrometers in compact devices for optical diagnostics and control of the optical properties of condensed scattering media. In addition, the goal was to describe in detail the features of the connection scheme of these microspectrometers and the necessary methods for correcting the initially obtained spectral dependencies *S*(λ,ρ*_k_*). The documentation of the microspectrometer manufacturer gives the main parameters and characteristics, which are determined according to the methods and technical requirements adopted at its enterprise. They are the property of the manufacturer and are not made available to users. Engineers developing portable diagnostic equipment have to create their own methods for correcting the obtained spectral dependencies in cases of non-standard use of microspectrometers. The conditions for their use in non-standard tasks may differ from the conditions for determining the parameters and characteristics at the manufacturer enterprise. Therefore, the authors hope that the detailed description of the features of the C12880MA and C11708MA microspectrometers and the microprocessor circuit for their connection, as well as the necessary methods for correcting *S*(λ,ρ*_k_*) given in the article, will accelerate the development and implementation of appropriate portable equipment for conducting the real-time optical control of fine materials and media.

## 2. Design Features of Hamamatsu Microspectrometers

Considering the prospects for introducing modern spectrometry technologies into everyday practice, the Hamamatsu Corporation has mastered the serial production of microspectrometers, which are distinguished by an extremely compact design with external dimensions of 20.1 × 12.5 × 10.1 mm^3^ (C12880MA) and 27.6 × 16, 8 × 13 mm^3^ (C11708MA). The optical scheme of these microspectrometers is standard with the use of an entrance slit and a concave focusing diffraction grating with a plane field of the formed image of the slit [25,26]. The registration of the radiation spectrum, which falls on the entrance slit and is then split by the diffraction grating, is carried out by a CMOS array of photodetectors. Concave diffraction gratings are formed by nano-printing. An entrance optical slit is integrated into the array substrate using etching technology.

The simpler architecture of the C11708MA array of photodetectors assumes the use of the “rolling shutter” mode. In this mode, the shift register of the spectrometer photodiode array at the negative edge of the measurement start signal ST starts the integration circuit of the photogenerated current generated by the first element of the photodiode array [26]. The multiplexer built into the shift register unit provides further sequential start (with a shift by 4 clock cycles of the synchronous signal CLK) of the photocurrent integration circuits of the second, the third, etc., up to the 256-th element of the photodiode array. The required value of the integration time τ is set, forming the required clock frequency *f_CLK_* ≤ 800 kHz. The reading of the video signal is carried out by alternately connecting (for the 4-clock-cycle synchronous signal CLK) the photocurrent integration circuits of the elements of the photodiode array to the integrating amplifier of the reading output stage. By increasing the value of the integrating capacitance at this stage from 1.4 pF to 4.8 pF by applying a low level to the Gain of the spectrometer, its sensitivity can be reduced by about 3.5 times [23]. The last stage of the array reading circuit forms a video signal, which is then fed from the Video output to repeaters on analogue operational amplifiers and then to the ADC of the microprocessor board.

The digital signal Ds registered by the ADC is usually divided by its integration time τ during further digital processing. This makes it possible to form a measured value *V* = *D_s_*/τ which is proportional to the photocurrent *I_fd_ = S_I_*(λ)⋅Φ(λ) formed in photodiodes due to the radiation flux Φ with a wavelength λ hitting their photosensitive areas, where *S_I_*(λ) is the spectral slope of the current conversion of photodiodes.

The architecture of the C12880MA microspectrometer array of photodetectors uses another more progressive accumulation and readout mode called the Global shutter. The exposure time or the photocurrent integration time τ is set the same for all light-sensitive elements of the array. Optimal for this mode is the use of an analogue photocurrent integrator in each photosensitive element. After the end of the exposure, the signal voltage from the output of each integrator is transferred with the help of electronic keys to the corresponding elements of the storage circuit, from where they are read out in turn and transferred to the Video bus during the next accumulation cycle. Such organization of the cycle of accumulation–reading of signals from the photodetector array allows to optimize the process of measuring spectra at long exposure times τ, since the process of reading signals practically does not affect the time of their accumulation.

## 3. Connecting Microspectrometers to a Personal Computer

To connect the microspectrometers under consideration to a computer via the USB2.0 interface, the Hamamatsu Corporation offers a standard board C11351 [25,26], which has inconvenient dimensions and high cost. Since modern microprocessors of the STM32 series have all the necessary components for connecting microspectrometers, we have developed the schemes for connecting the C12880MA and C11708MA to the microprocessor and USB port of the computer. During the development, the goal was to miniaturize the design of the spectrometer by minimizing the number of necessary additional electronic components taking into account the architecture of the photodetector arrays used in spectrometers. Among the solutions available on the world market, the STM32 family of microcontrollers based on the ARM Cortex-M core developed by STMicroelectronics has become widespread due to the easy availability of the proposed solutions, low prices, and the availability of a convenient environment for their programming. The STM32F103C8T6 ARM STM32 (Blue pill) mini-debug board based on a powerful STM32F103C8T6 chip has the smallest dimensions. It is powered by the computer’s USB port and has all the necessary connectors and interfaces for connecting to a computer and other devices, including a programmer. The small dimensions of this board (10 × 23 × 54 mm^3^) and the ample opportunities it offers make it possible to create small-size spectrometric devices on its basis.

### 3.1. Features of the C12880MA and C11708MA Connection Diagrams

The developed connection diagram of the considered spectrometers to the Blue pill board is based on the Hamamatsu-recommended standard connection diagram of the necessary external components to the spectrometer [25,26]. In order to transmit the video signal to the ADC input of the microprocessor, which is formed at the Video output of the considered spectrometers, two serially connected broadband analogue repeaters based on the MCP6022-I/SN dual operational amplifier are used. Since the video signal range of the C12880MA microspectrometer is from 0.3 V to 4.3 V, a resistive signal divider is used in the circuit at the output of the first analogue repeater, which ensures that the video signal range is matched to the microprocessor ADC conversion range from 0 V to 3.3 V. There is no resistive divider in the C11708MA connection diagram, since the range of the video signal of this micro-spectrometer is narrower, only from 0.15 V to 3.3 V.

In the developed circuit, the microspectrometers are powered (+5 V) from the USB port. The Blue pill board used contains a built-in stabilized microprocessor power supply with a voltage of +3.3 V, which provides the necessary conditions for stable operation of the built-in 12-bit ADCs. To match the levels of digital signals used in the microprocessor (from 0 V to +3.3 V) and microspectrometers (from 0 V to +5 V), the SN74ACT04DR level converter is used. The microspectrometers are powered from the USB power bus, where the impulse noise occurs with the exchange of data packets. Therefore, microspectrometers with repeaters based on the MCP6022-I/SN amplifier are powered through an LC Pi low-pass filter.

### 3.2. Specificity of the Software Used

The developed software has two levels: (1) the lower one, which is the microprocessor microprogram, which forms the signal diagrams that set the operating modes of the microspectrometer, frame reading, their averaging and transmission to the upper level; (2) the upper one, providing the subsequent processing of the obtained spectra and their visualization.

The developed microprogram provides the ability to continuously control the operation of the microspectrometer and to promptly respond to the microprocessor’s requests to the control computer via the USB bus. Due to the small difference between the clocking frequencies of the microprocessor (72 MHz) and CLK of the C12880MA microspectrometer (4.8 MHz), it is impossible to programmatically generate the stable parameters of timing diagrams using the HAL library. Therefore, control signal diagrams are generated in hardware, which ensures the achievement of the maximum allowable clock frequency CLK of the C12880MA spectrometer. A unique cascade mode of operation of timers is used, in which the hardware timers control the start, clocking, dynamic change of parameters of other timers, ADC, and states of the microprocessor output ports. Before measuring the frame, the microprocessor only carries out the initial setting of the necessary blocks (four timers, an ADC and a DMA block) and is no longer engaged in timing, setting the exposure duration and reading the frame. The applied solution ensured stable operation of the C12880MA spectrometer in a wide range of frame exposures from 12 μs to tens of seconds.

The C12880MA uses a global shutter mode that allows different CLK frequencies for exposure and frame reading. During the exposure, a high CLK frequency is used, which provides a minimum integration time, and a smaller step for its setting. Since the maximum possible conversion frequency of the ADC of the microprocessor is about 5 times lower than the used CLK frequency of microspectrometers, the CLK frequency is reduced to 800 kHz when the video signal is subtracted.

Since the C11708MA microspectrometer uses the rolling shutter mode, a different control signal pattern is generated without using CLK switching. The exposure of the next frame is carried out during parallel reading of the previous frame. For this microspectrometer, the maximum possible constant clock frequency CLK of 800 kHz is used and the ADC is clocked at a frequency of 200 kHz. Since the sensitivity of the C11708MA is much lower compared to the C12880MA, the minimum allowable exposure time is 1.29 ms. The Gain signal has been added to the control signal diagram, which is set to the High gain state by default.

### 3.3. Reduction of the Influence of the Nonhomogeneity of the Photodetector Array Dark Counts

A simplified expression for the voltage of the signals generated by the photosensitive elements of the array of photodetectors of the considered spectrometers, which are then transmitted through the reading unit and repeaters to the ADC input, is the following:

(1) $$U_{i}\left(\tau \right)=U_{bi}+\frac{i_{phi}+i_{di}}{C_{c}}\tau +n_{i}\left(t\right),$$

where *U_bi_* is the DC bias voltage of the video signal for the *i*-th element of the array that depends on the temperature of the internal cavity of the spectrometer and the bus voltage +5 V of the USB port; *i_phi_* and *i_di_* are photocurrents and dark currents generated in the light-sensitive cells of the spectrometer photodiode arrays, respectively, *C_c_* and τ are correspondingly the capacitance of the signal integration circuit and the exposure time.

The *U_bi_* or baseline values are easily determined by averaging the spectra registered with the spectrometer slit closed, i.e., ${\bar{U}}_{bi}={\bar{U}}_{i}\left(\tau \right)-\left(i_{di}/C_{c}\right)\tau -{\bar{n}}_{i}\left(t\right)$. Since the average value of the total noise, including the dark current noise, thermal noise, and the noise of the photodiode array reading unit, tends to zero upon averaging and the contribution of the dark current at small τ is insignificant, we obtain ${\bar{U}}_{bi}\approx {\bar{U}}_{i}\left(\tau \right)$.

**Figure 1:**
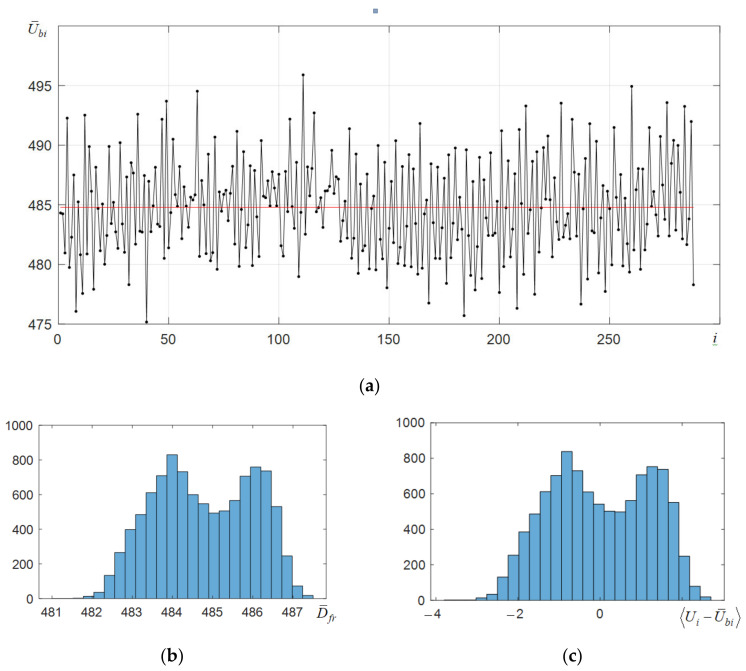

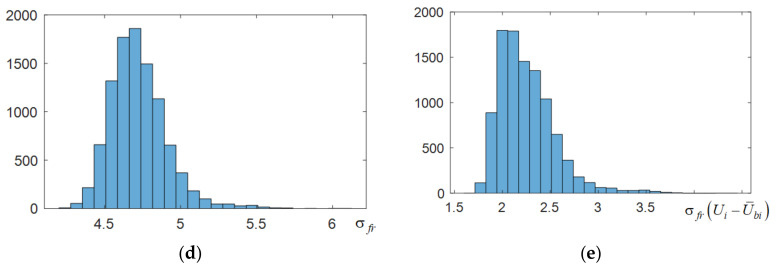
Dependence of the averaged (over 10,000 frames) dark counts on the number *i* of the photodiode array element of the microspectrometer C12880MA (**a**), histograms of distribution of mean values without (**b**) and after (**c**) subtracting the average baseline ${\bar{U}}_{bi}$, histograms of distribution of standard deviation without (**d**) and after (**e**) subtracting the average baseline ${\bar{U}}_{bi}$ by frames.

The C12880MA microspectrometer has a noticeable baseline inhomogeneity. As seen from Figure 1a, the maximum deviation of the values of dark samples ${\bar{U}}_{bi}$ averaged over 10,000 frames from the average value (shown by the red solid line) is 6 ADC levels. Due to the revealed non-homogeneity of the baseline and the presence of noise, the root-mean-square deviation (RMSD) of the obtained dark counts approaches 5 units, which is illustrated by the histogram in Figure 1d. When using the option of subtracting the baseline from the spectra, the RMSD values of dark counts (Figure 1e) are reduced almost twice. When normalizing to the average value of dark counts (${\bar{U}}_{b}$= 485), the RMS of dark counts decreases from 1% to 0.47%. The deviation of the shape of the RMS histograms (obtained by subtracting the baseline) from the Gaussian distribution (Figure 1e) indicates the presence of impulse noise in the registered dark counts. This impulse noise is generated both by the photodiode array of the C12880MA spectrometer, which includes 288 elements, and by the 12-bit ADC built into the microprocessor. Due to their influence, the bias histograms of the dark counts from the baseline (Figure 1c) are stretched and have 2 modes. To eliminate the influence of the revealed non-homogeneity, the developed software has options for determining the averaged baseline and subtracting it from the measured spectra. Their use makes it possible to reduce both the inhomogeneity of the averaged baseline ${\bar{U}}_{bi}$ and RMSD of the obtained digital counts of the C11708MA microspectrometer to approximately 1 ADC level, which is illustrated by the histograms in Figure 1d,e. Note that the non-Gaussian shape of the RMS distribution histogram for the C11708MA also indicates the presence of impulse noise. The baseline heterogeneity of the C11708MA microspectrometer is less than that of the C12880MA one. Its subtraction from the measured spectra leads to the histograms similar to those shown in Figure 1d,e.

## 4. Improvement of Equivalent Spectral Resolution Using Wiener Filtering

As noted above, a feature of the analysis of the optical properties of condensed media is the need to obtain spectra with the least distortion, since it is necessary to solve ill-posed inverse problems. Due to the small size of the C12880MA and C11708MA spectrometers, their spectral resolution *r*(λ) is limited and is about 9 nm and 14 nm, respectively [22,23]. Although in many practical problems of analyzing the properties of condensed media this resolution may be sufficient, the problem of analyzing the possibilities of improving the equivalent spectral resolution of the considered microspectrometers obtained by using digital processing of the recorded spectra remain urgent [24].

In their documentation, manufacturers of small-sized spectrometers usually cite the resolution *r*, which is determined at half-height of the instrumental function. Let us note that the instrumental function *h(*λ*)* is the response of the spectrometer to monochromatic radiation, which is usually normalized so that the area under it is equal to unity. The shape *h(*λ*)* depends on a number of spectrometer parameters, including the slit width, beam path, dispersion of the concave diffraction grating, and its shape.

Reconstruction of the shape of the spectrum distorted by a spectrometer with a finite resolution in the general case [29] is reduced to solving the following equation containing a convolution integral:

(2) $$\int_{\lambda_{L}}^{\lambda_{R}}S_{r}\left(\lambda \right)h\left(\lambda -\xi \right)d\xi +n\left(\lambda \right)=S_{m}\left(\lambda \right),$$

where *S_r_*(λ) is the true radiation spectrum, ξ is the shift of the instrumental function of the spectrometer along the axis of λ values, λ*_L_* and λ*_R_* are the left and right boundaries of the range of determination of the instrumental function, in which it is different from zero, respectively, *n*(λ) is the noise caused by the radiation noise of dispersed radiation and the intrinsic noise of the spectrometer, *S_m_*(λ) is the spectrum measured by the spectrometer. In the presence of noise, Equation (2) does not have an exact solution; therefore, it is solved approximately using additional criteria for evaluating the approximation. If we use the minimum of the mean square of the error as a criterion and use only linear processing methods, then the optimal solution (2) is linear filtering using the Wiener filter [29,30,31,32]:

(3) $$S_{r}^{*}\left(\lambda \right)=F^{-1}\left\{H_{W}\left(\omega_{\lambda }\right)\cdot F\left[S_{m}\left(\lambda \right)\right]\right\},$$

where the operators F and F^−1^ represent the direct and inverse Fourier transform, respectively, and the transfer function of the Wiener filter in the frequency domain has the following form:

(4) $$H_{W}\left(\omega_{\lambda }\right)=\frac{H^{*}\left(\omega_{\lambda }\right)}{{\left|H\left(\omega_{\lambda }\right)\right|}^{2}+P_{N}\left(\omega_{\lambda }\right)/P_{S}\left(\omega_{\lambda }\right)},$$

where *H*(ω_λ_) is the Fourier transform of the instrumental function *h*(λ), ω_λ_ = 2π*f*λ is the cyclic frequency, where *f_λ_* has a dimension inverse to the period Λ of the harmonic oscillation in the spectrum, i.e., *f_λ_* = 1/Λ 1/µm, H*(ω_λ_) is the complex conjugate dependence, *P_S_*(ω_λ_) and *P_N_*(ω_λ_) are the spectral energy densities of the registered spectrum and its total noise (generated by intrinsic noise of spectrometer and radiation noise of the registered radiation), respectively. Note that the direct and inverse Fourier transforms used in expression (3) have the properties of linearity. Therefore, the shape of *S*^∗^*_r_*(λ) as a result of Wiener filtering may differ from the initial spectrum *S_m_*(λ), but the linear dependence of *S*^∗^*_r_*(λ) on the intensity of radiation incident on the entrance slit of the spectrometer is preserved.

The main difficulty in forming the transfer function of the Wiener filter *H_W_*(ωλ) is the need to know the *P_N_*(ω_λ_)/*P_S_*(ω_λ_) ratio. Investigations of the intrinsic noise of microspectrometers C12880MA and C11708MA have shown that they correspond to theoretical concepts, i.e., they consist of the sum of the noise of the reading unit of the used photodiode arrays, as well as the shot noise of the thermally generated dark current of the photodiodes and the radiation or photon noise of the dispersed radiation, which obey the Poisson distribution. The noises of the elements of the used photodiode array do not depend on each other, that is, they are not correlated with each other. The total spectrum of the noise of the reading unit and the thermally generated dark current of the photodiodes in the frequency range is approximately uniform, i.e., corresponds to the spectrum of white noise. Since the contribution of the considered noise to the obtained spectra is usually reduced by using the appropriate values of the exposure time τ and accumulating the frames, the resulting dependences of the *P_N_*(ω_λ_)/*P_S_*(ω_λ_) ratio are usually noticeably less than 10^−4^, i.e., they practically do not affect the behavior of the transfer characteristic of the Wiener filter in the low-frequency region. Since in this region the *P_N_*(ω_λ_)/*P_S_*(ω_λ_) ratio weakly depends on the frequency ω_λ_, that is, it is practically a constant, it is usually replaced by a constant parameter β. Typical values of β usually lie in the range from 10^−8^ to 10^−2^ [29]. In practice, such minimum value of β is experimentally selected at which the corrected spectrum does not yet have parasitic fluctuation oscillations.

## 5. Equipment for Spectra Measurement

**Figure 2:**
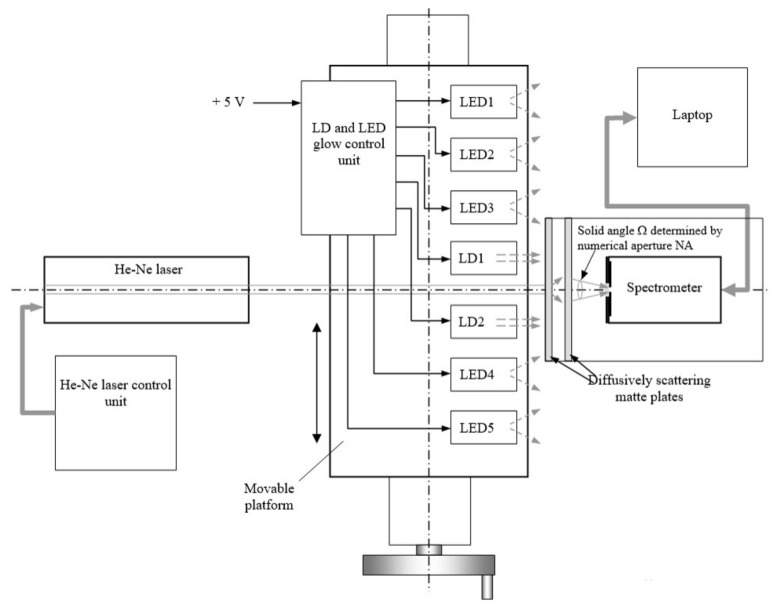
Functional diagram of the equipment for measuring spectra of the eight radiation sources used.

When obtaining the instrumental function of the spectrometer *h*(λ), the illumination of the entrance slit of the spectrometer by laser radiation with a long coherence length is optimal. Therefore, the equipment must contain at least one stabilized gas laser. It is also advisable to include laser diodes in it in order to find out the possibility of their use when measuring *h*(λ). Moreover, in order not to distort the shape of the instrumental function, it is necessary to use a measurement circuit that is equivalent to the circuit for recording spectra *S*(λ) of diffusely scattered radiation. The numerical aperture NA of the considered spectrometers is equal to 0.22 [22,23], and the corresponding angle α = 2⋅arcsin(NA) ≈ 26°. Radiation fluxes in the solid angle Ω shown in Figure 2 participate in the formation of the spectrum *S*(λ). The value of Ω is determined by the numerical aperture NA and the length of the spectrometer slit. To create an extended source of coherent radiation in the experimental setup, one should use diffuse-scattering elements that are illuminated by a narrow- collimated laser beam. In order to evaluate the efficiency of the proposed correction of the obtained spectral dependencies in the visible and near-infrared regions of the spectrum, a set of LEDs with different radiation wavelengths can be used. The radiation of LEDs is incoherent and non-polarized; moreover, the intensity of their radiation is easy to control. Therefore, based on the above considerations, we used equipment for measuring the spectra of the radiation sources used, which is shown in Figure 2.

The equipment is assembled on an optical bench and contains a device for attaching a stabilized He-Ne laser LGN-303 with the necessary alignment devices, a platform for placing spectrometers, two diffusely scattering white Plexiglas plates, and a movable platform on which two laser diodes (LD1 and LD2) with maximum emission wavelengths of 443.5 nm and 789.7 nm, respectively, as well as five LEDs (LED1–LED5) with different maximum emission wavelengths in the range from 450 to 936 nm are located. The power of collimated radiation of the used He-Ne laser and the laser diodes used is 1.5 mW and 2–3 mW, respectively. To stabilize the radiation power of laser diodes and LEDs, a set of adjustable current generators installed in the LD and LED glow control unit was used. Since the divergence of the light radiation generated by the LEDs is large, the power of their radiation was set to approximately equal 5 mW.

The use of diffusely scattering plates makes it possible to form a rather extended spot of scattered radiation at the exit of the second plate with an approximately uniform intensity in its center. In order for the spot size with uniform illumination to exceed the field of view of the used spectrometer, its entrance slit was set exactly on the optical axis at a distance of about a millimeter from the surface of the last scattering plate. The He-Ne laser is promptly installed and adjusted so that its radiation propagates along the axis on which the entrance slit of the spectrometer is located. In order to obtain the spectral response of the semiconductor emitter *S*(λ), the emitter was shifted onto the optical axis of the equipment with the help of a movable device. Then, its position was adjusted by slightly shifting the movable platform with emitters and visual observation of the registered spectrum on the laptop screen. Turning the movable device, we achieved the maximum amplitude of the observed spectrum.

To compare the spectra registered by microspectrometers with the spectra obtained with a resolution of about 5 nm, the spectra of all used radiation sources were registered also with an AvaSpec 2048L spectrometer with an entrance slit width of 100 µm. Then, the spectrometer C12880MA registered the spectra of all 3 lasers and 4 LEDs (LED–LED4), since the emission spectrum of LED5 with λ*_max_* = 936 nm lies outside the spectral range of C12880MA. The infrared spectrometer C11708MA registered the spectra of the He-Ne laser and LD2, as well as the spectra of three LEDs (LED3–LED5). To eliminate the effect of background illumination during measurements, the background spectra obtained when the radiation sources were turned off were also registered. During further processing, the background spectra were subtracted from the spectra of the corresponding radiation sources. The resulting radiation spectra, together with the background ones, were registered in files on a laptop disk with the necessary accompanying information (time of spectra recording, exposure time, the number of spectra recordings, etc.). During further processing, the averaging of the registered spectra was used to increase the signal-to-noise ratio.

## 6. Research Methodology and Results

When creating a research methodology, the goal was to provide a comparison of the results obtained for three types of spectrometers in order to facilitate understanding the reasons for the differences in their parameters. Therefore, after obtaining the initial spectra of the radiation sources used, they were processed using a unified technique for unambiguous interpretation of the results.

### 6.1. Correction of the Registered Spectra, Eliminating the Influence of Nonlinearity of the Light Characteristic of the Spectrometer

The nonlinearity of the light characteristic, i.e., the dependence of the counts of the analogue-to-digital converter (ADC) of the spectrometer *D*(*λ_i_*) on the radiation flux Φ(λ*_i_*) illuminating its entrance slit, leads to visually weakly noticeable distortions of the obtained spectra. However, upon further processing, these distortions appear in the form of a deviation of the obtained results from the true ones. Therefore, at the first stage of the applied method, the corresponding correction of the registered spectra was carried out.

The spectral sensitivity of spectrometers based on photodiode arrays is mainly determined by the spectral dependencies of the sensitivity of the array used and the blaze angle of the diffraction grating. Since they are nonuniform, the relative spectral sensitivities *s*(λ) of the C12880MA and C11708MA spectrometers, which are given in [22,23], decrease to the boundaries of the operating spectral range. To eliminate the influence of non-uniformity *s*(λ), such spectrometers are usually calibrated in advance by reference emitters [28]. In the absence of calibration, the normalization of the obtained diffusion reflectance spectra to the reflectance spectra of standard scattering white samples *S_W_*(λ*_i_*) is often used. These reference scatterers are usually included in the delivery set, for example, WS-2 by Avantes (Holland). Since the light characteristics are formed by the path for converting photogenerated charges into digital counts, they, after normalisation to *S_W_*(λ*_i_*), i.e., in a relative form, weakly depend on the wavelength.

**Figure 3:**
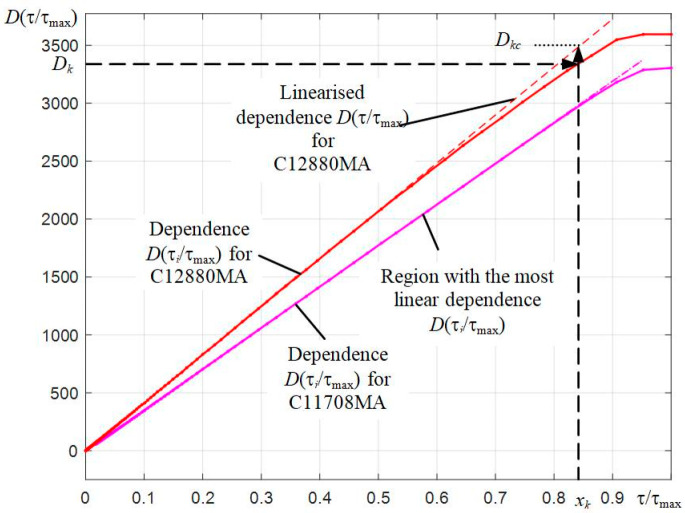
Measured light characteristics of C12880MA and C1170MA microspectrometers, normalized along the abscissa axis, and an illustration of the process of their linearization.

The ability to automatically set the frame exposure time τ allows to simplify and automate the process of determining the light characteristics. The presence in the spectrometer of a photodiode array with a controlled value of τ makes it possible to change the recorded digital counts *D* at a constant value of the radiation flux Φ, illuminating the entrance slit of the microspectrometer, by changing τ. The relative light characteristics *D*(τ/τ*_max_*) of the C12880MA and C11708MA spectrometers with their switching circuit used were determined by recording the LED spectra with a smooth automatic increase in the exposure time τ from the minimum possible value until the full saturation of the light characteristic at the wavelength λ*_i_* = λ*_max_* corresponding to the maximum of the spectrum of the radiation source used. The radiation power of the source used was kept constant. The values of the obtained digital counts *D* at the ADC output are proportional to the photocurrent *i_ph_*, which is generated in the photosensitive elements of the photodiode array, and the frame exposure time τ, i.e., *D*(τ) ~ *i_ph_*⋅τ. Since the exposure time τ in the developed connection circuit is set with a relative error of 10^−5^, and the photocurrent *i_ph_* at a constant power of the radiation source is constant *i_ph_* = const, a linear dependence of the generation of the resulting photogenerated charge *q* = *i_ph_*⋅τ is provided. The nonlinearity of the relative light characteristic, i.e., the deviation of the *D(q)* dependence from the linear law, is in the deviation of the obtained dependence *D*(τ/τ_max_) from the straight line *D* = *b*⋅*x*, as the charge *q* approaches saturation, i.e., at τ → τ_max_. The dependencies *D*(τ/τ_max_) obtained when using the radiation of red LEDs for C12880MA and an infrared LED with a maximum emission at a wavelength of 757 nm for C11708MA are shown in Figure 3. For each exposure value, 16 spectra were registered with their further averaging.

Analyzing the graphs in Figure 3, we see that the measurement mode with a Global Shutter used in the photodiode line of the C12880MA spectrometer has the worst linearity of the *D*(τ/τ*_max_*) dependence, and a noticeable nonlinearity is observed at large values of *D*. The Rolling Shutter mode used in the array of photodiodes of spectrometer C111708MA provides better linearity.

To correct the influence of this nonlinearity, an algorithm was introduced into the software based on the use of predetermined relative dependence *D*(*x_k_*) = *D*(τ*_k_*/τ*_max_*) for each of the spectrometers. This characteristic is approximately the same for all elements of the photodetector array, i.e., does not depend on the wavelength λ*_i_*. As seen from Figure 3, the *x_k_* values for the corresponding dependence *D*(*x_k_*) are found from the obtained digital count *D_k_* by interpolation. Next, the *x_k_* values are multiplied by the slope coefficient b of the *D*(*x_k_*) segment with the most linear dependence. This gives the corrected values *D_kc_* = *b*⋅*x_k_*, which will lie on the straight line. The extrapolation of the linearized *D_kc_* dependencies shown by dashed lines passes through the origin of the coordinates. The calculated *b* values were 4141.5 for C12880MA and 3545.7 for C11708MA.

### 6.2. Transition to Linear Scale for Samples with a Smaller Sampling Interval

The next step in investigating the possibility of using the discussed microspectrometers should be the conversion of the scale of their counts by wavelengths. In microspectrometers based on photodiode arrays, it is nonlinear, and with a decrease in the used optical path length of the rays, this nonlinearity increases. In addition, the number of elements in the array of photodetectors of microspectrometers is small: 288 elements for C12880MA and 256 elements for C11708MA. Therefore, to improve the visual perception of the obtained spectra, it is necessary to interpolate them on a smaller scale of counts, i.e., with a smaller sampling interval λ. Moreover, to simplify further transformations, it is expedient to form a new linear scale of counts.

**Figure 4:**
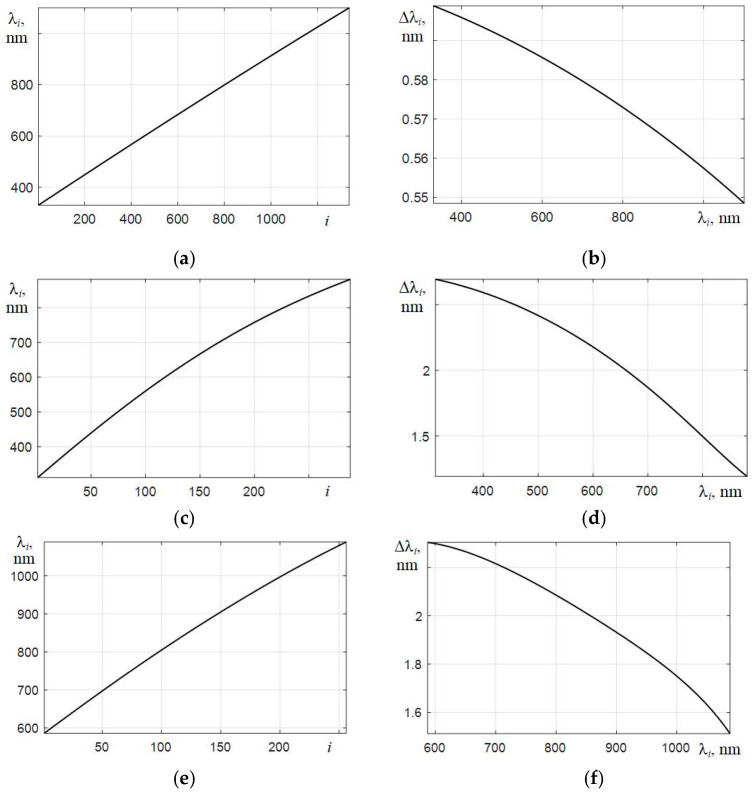
Counts λ*_i_* vs. the element number of the array of photodetectors *i* and the interval Δλ*_i_* between the counts for the AvaSpec spectrometer (**a**,**b**) and microspectrometers C12880MA (**c**,**d**) and C11708MA (**e**,**f**), respectively.

**Figure 5:**
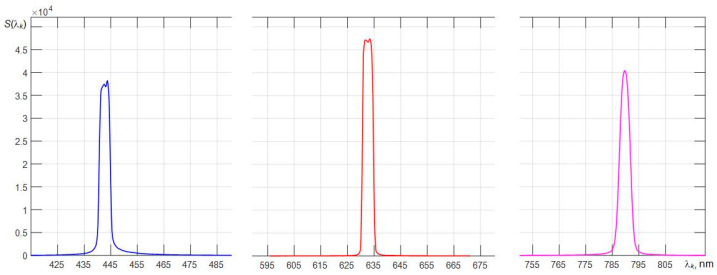
Spectra of three lasers interpolated on a fine scale of counts (4096 counts were used in the range from 330.4 to 1100 nm) registered with an AvaSpec 2048L spectrometer.

Figure 4 shows the dependencies of the counts of wavelengths λ*_i_* on the number of the element of the array of photodetectors for the three types of spectrometers used. As expected, the greatest nonlinearity is characteristic for the C12880MA with the shortest optical path [26]. Since the method for improving the equivalent spectral resolution *r* is based on the use of the Wiener filter in calculating its transfer characteristic, one has to use a fast Fourier transform. Therefore, when forming a new scale of samples λ*_k_*, it is advisable to use spline interpolation of the obtained spectra with the number of samples equal to 2*N*. To give visual smoothness to the interpolated spectra, which are displayed on the monitor screen, it is better to choose the *N* value in the range from 11 to 13. In this case, the dependences of the interpolated spectra of laser radiation sources become sufficiently detailed, which is illustrated by the graphs in Figure 5 obtained with an AvaSpec spectrometer. It is clearly seen that the smallest width of the base of the spectral peak is characteristic of the spectrum of a He-Ne laser with a long radiation coherence length. For the spectra of laser diodes, the bases of the corresponding peaks are broadened mainly due to the contribution to the spectra of their spontaneous emission.

The documentation for the AvaSpec 2048L indicates that its spectral resolution when using a diffraction grating with a period of 300 lines/mm and an entrance slit width of 100 μm is 4.8 nm. The values of the resolving power of the AvaSpec spectrometer calculated by us at the half-height of the spectral peaks of laser radiation turned out to be equal: *r* = 4.3 nm at λ = 443.7 nm, *r* = 4.4 nm at λ = 632.8 nm, and *r* = 4.4 nm at λ = 789.7 nm, which is close enough to the *r* resolution declared by their manufacturer. The shape of the registered spectral peak for a He-Ne laser is close to the theoretical one, i.e., rectangular. Indeed, a concave diffraction grating focuses the diffracted beams in the plane of location of the light-sensitive elements of the CMOS photodiode array, forming an image of a uniformly illuminated entrance slit. Therefore, a rectangular peak is recorded. Note that instead of a plateau at the peak tops of the spectra of the He-Ne laser and LD1, the oscillations are observed due to the effect of the speckle structure of the image of the entrance slit of the AvaSpec spectrometer.

During operation of spectrometers, a shift in the reading scale may occur, which must be corrected. The stability of the lasing wavelength of the LGN-303-stabilized He-Ne laser used in the equipment is not worse than 10^−8^; therefore, its radiation can be considered a reference one with λ = 632.819 nm. For each spectrometer, the He-Ne laser wavelength according to its scale λ was determined from the corresponding spectral response according to the following expression:

(5) $$\lambda_{\mathrm{He}-\mathrm{Ne}}=\int_{\lambda_{L}}^{\lambda_{R}}\lambda S_{\mathrm{He}-\mathrm{Ne}}\left(\lambda \right)d\lambda /\int_{\lambda_{L}}^{\lambda_{R}}S{\left(\lambda \right)}_{\mathrm{He}-\mathrm{Ne}}d\lambda ,$$

where λ*_L_* and λ*_R_* are the left and right boundaries of the integration range, and *S*_He-Ne_(λ) are the laser spectra registered by the spectrometers. The measured offsets of the reference scale were Δλ*_ws_* = −0.07 nm for the AvaSpec 2048L spectrometer, Δλ*_ws_* = −3.12 nm for the C12880MA, and Δλ*_ws_* = −2.39 nm for C11708MA. During further analysis, the count scales of λ of each spectrometer were corrected, that is, shifted to the right by the corresponding value Δλ.

### 6.3. Registered Instrumental Functions of Spectrometers

To compare instrumental functions with each other, it is necessary to normalize them. The most obvious normalization follows from the condition that a narrow initial radiation spectrum of a He-Ne laser, which in the case under consideration can be represented as a δ-function, is registered by spectrometers in the form of spectral peaks of a certain shape, the area under which should be the same for all used spectrometers. This area can be reduced to unity by the simplest normalization:

(6) $$h\left(\lambda_{k}\right)=\frac{S_{\mathrm{He}-\mathrm{Ne}}\left(\lambda_{k}\right)}{\Delta \lambda \sum_{k}S_{\mathrm{He}-\mathrm{Ne}}\left(\lambda_{k}\right)}.$$

**Figure 6:**
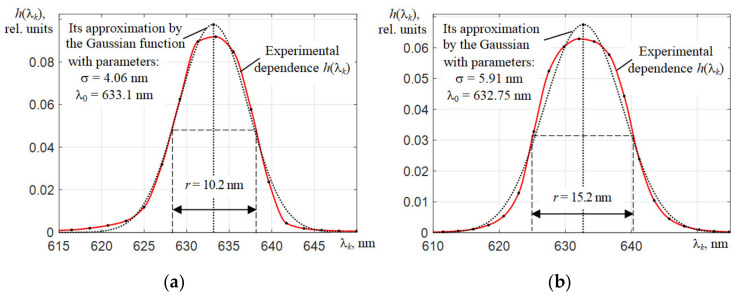
Instrumental functions of spectrometers C12880MA (**a**) and C11708MA (**b**) (solid lines with dots), obtained by illuminating their entrance slits with scattered radiation from a He-Ne laser and their approximation by the Gaussian function (dotted lines) after correcting the shifts of their wavelength λ*_k_* count scales.

The measured instrumental function *h*(λ*_k_*) of the C12880MA microspectrometer when illuminated its entrance slit with dimensions of 50 × 500 μm^2^ by the scattered radiation of a He-Ne laser deviates from its approximation by a Gaussian dependence:

(7) $$h_{G}\left(\lambda_{k}\right)=1/\left(\sqrt{2\pi }\sigma \right)\exp\left[-{\left(\lambda_{k}-\lambda_{0}\right)}^{2}/\left(2\sigma^{2}\right)\right],$$

at σ = 4.06 nm and λ_0_ = 633.1 nm, which is illustrated in Figure 6a. When approximating, the values of σ and λ_0_ were fitted using the least squares method so that the areas under the curves *h*(λ*_k_*) were the same. In addition to the faster decay of the wings of the *h*(λ*_k_*) dependence as compared to the approximation by the Gaussian function *h_G_*(λ*_k_*), there is a slowly decreasing bias, the origin of which still needs to be clarified. Most likely, it is caused by the shape of the surface of the concave diffraction grating formed by nano-printing.

The somewhat large dimensions of the C11708MA spectrometer and the dimensions of its entrance slit 75 × 750 μm^2^ contribute to the approximation of the shape of its instrumental function to a trapezoidal one. The spectral resolution *r* of the C11708MA spectrometer should be worse than the C12880MA, since the entrance slit width is 1.5 times larger. The measured values of the spectral resolution of *r* = 10 nm and *r* = 15.2 nm of the C12880MA and C11708MA spectrometers, respectively, turned out to be somewhat worse than those declared by their manufacturer (about 9 nm and 14 nm [22,23]). This may be due to the difference in the values of the solid angles in which the radiation from extended sources during measurements was directed to the entrance slit of the spectrometers.

**Figure 7:**
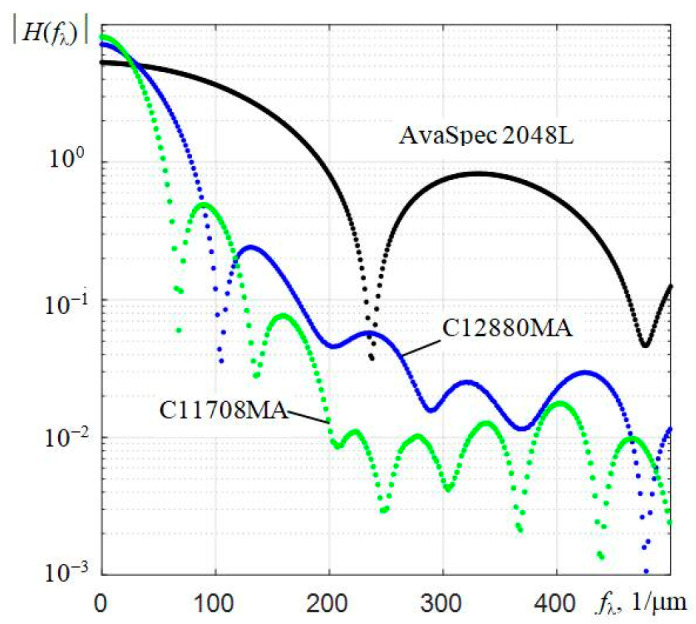
Moduli of the components of the Fourier transforms of the instrumental functions |*H*(*f*_λ_)| of three types of spectrometers in the frequency *f*_λ_ range from 0 to 500 1/μm.

Fourier transform moduli |*H*(*f*_λ_)| of interpolated instrumental functions *h*(λ*_k_*) of three investigated spectrometers in the frequency range from 0 to 500 1/μm are shown in Figure 7. The used dependences *h*(λ*_k_*) were obtained by illuminating the entrance slits of the spectrometers with the scattered radiation of a He-Ne laser. When calculating |*H*(*f*_λ_)|, the corresponding ranges of the scales of counts by wavelengths were set and the discrete fast Fourier transform (FFT) was applied to the array *h*(λ*_k_*). For the AvaSpec 2048L spectrometer, the range was 330.5–1100 nm, and for the C12880MA and C11708MA, the ranges were 314.7–885.1 nm and 588.5–1091.3 nm, respectively. It is recommended to use dependencies containing 2*^N^* counts when conducting FFT. Therefore, when determining |*H*(*f*_λ_)|, the interpolation *h*(λ*_k_*) was applied using a small scale of wavelength values λ. In order to obtain sufficient resolution of the modules of the components of the Fourier transforms |*H*(*f*_λ_)| of the instrumental functions of the studied spectrometers in the range of *f*_λ_ values from 0 to 500 1/μm (shown in Figure 7 by separate dots), the value of *N* = 12 was chosen, i.e., 2^12^ = 4096 counts were used. Since the spectral ranges of spectrometers are different, the intervals Δ*f*_λ_ between the components of the |*H*(*f*_λ_)| dependencies in Figure 7 have different meanings. The upper values of max*f*_λ_ of the frequency range were calculated on the basis of the corresponding intervals Δλ of small linear count scales λ*_k_*, i.e., max*f*_λ_ = 1/(2⋅Δλ).

The shape of the |*H*(*f*_λ_)| modulus for the AvaSpec 2048L spectrometer resembles the spectrum of a rectangular pulse. The first lobe of this modulus |*H*(*f*_λ_)| has components up to the frequency *f*_λ_ ≈ 240 1/μm, and in the case of the inverse Fourier transform, they practically form the shape of the instrumental function *h*(λ). As expected, the Fourier transforms of the instrumental functions of the C12880MA and C11708MA spectrometers have much narrower lobes and a steeper decrease, which reflects their poorer spectral resolution. If we compare the spectral resolution of spectrometers in terms of the width of the first lobe of the Fourier transform of the instrumental functions *h*(λ), we can say that it is about two times and three times worse for C12880MA and C11708MA, respectively, than for AvaSpec.

The standard FFT algorithm calculates the coefficients that differ from the coefficients of the expansion of the dependence *h*(λ*_k_*) in the Fourier series:

(8) $$c_{n}=\frac{1}{\Lambda }\sum_{k}h\left(\lambda_{k}\right)e^{-j\omega_{\lambda n}\lambda_{k}}\Delta \lambda =\frac{\Delta \lambda }{\Lambda }\sum_{k}h\left(\lambda_{k}\right)e^{-j\omega_{\lambda n}\lambda_{k}},$$

by the absence of the factor Δλ/Λ, where Λ = λ*_R_* − λ*_L_* is the spectral range of the spectrometer. Therefore, for the sum $\sum_{k}h\left(\lambda_{k}\right)=1$, the first element *C*_0_ of the direct FFT is equal to one. Since the normalization was used so that the area under the spectral peak was equal to one $\sum_{k}h\left(\lambda_{k}\right)\cdot \Delta \lambda =1$, the zero spectral components |*H*(*f*_λ_ = 0)| of the obtained dependencies (Figure 7), that is, the sums $\sum_{k}h\left(\lambda_{k}\right)$, turned out to be equal to 1/Δλ, where Δλ is given in nm.

### 6.4. Formation of Transfer Characteristics of the Wiener Filter

If it is necessary to improve the equivalent spectral resolution of microspectrometers, then it is possible to use Wiener filtering. The optimality criterion in the formation of the frequency transfer characteristic of the filter (4) can be the best coincidence of the registered spectrum with the spectrum obtained by the spectrometer with the required resolution, and the minimum presence of false high-frequency oscillations in it. The selection of the parameter β, which is substituted in (4) instead of the ratio *P_N_*(ω_λ_)/*P_S_*(ω_λ_), is inconvenient in practice, since samples of spectra with the required resolution are usually absent. It is more practical to use an expression that approximately describes the *P_N_*(ω_λ_)/*P_S_*(ω_λ_) ratio.

The total noise of the microspectrometer is determined by the noise of the unit for reading and converting the signal into digital counts, as well as the shot noise of the dark current of the photodiode array and the radiation noise of the dispersed radiation. Shot and radiation noises are described by the Poisson distribution, for which the variance of the spread of the number of registered charge carriers is equal to their average number ${\bar{n}}_{e}$ accumulated during the exposure, i.e., $\sigma_{e}^{2}={\bar{n}}_{e}=\tau \left(i_{ph}+i_{d}\right)/e$ where *e* is the electron charge. Passing from charges to digital counts of the ADC, the total variance of counts for the *i*-th element of the photodiode array can be described by the following expression:

(9) $$\sigma_{\Sigma }^{2}\left(\lambda_{i}\right)=\sigma_{read}^{2}+k_{qD}\left[i_{ph}\left(\lambda_{i}\right)+{\bar{i}}_{d}\right]\tau =\sigma_{read}^{2}+k_{qD}{\bar{i}}_{d}\tau +k_{qD}i_{ph}\left(\lambda_{i}\right)\tau =\sigma_{read}^{2}+k_{qD}{\bar{i}}_{d}\tau +S_{m}\left(\lambda_{i}\right)$$

where $\sigma_{read}^{2}$ is the variation of the spread of ADC counts caused by the noise of the reading unit, *k_qD_* is the conversion factor of the registered charges into digital counts, ${\bar{i}}_{d}$ is the average value of the dark current of the photodiode array. Reading noises, as well as dark current noises of the *i*-th elements of the photodiode array, do not depend on each other, i.e., they are uncorrelated. Therefore, the Fourier transform of their autocorrelation function is similar to the power spectral density of white noise, i.e., on average, it has a constant *P_rd_* value. Then, the Fourier transform of the total noises *P_N_*(ω_λ_) will contain a component in the form of a constant value *P_rd_* determined by the first two terms in (9), as well as the modulus of the Fourier transform *G*(ω_λ_) of the registered spectrum *S_m_*(λ). This allows us to write the ratio *P_N_*(ω_λ_)/*P_S_*(ω_λ_) in the following form:

(10) $$\frac{P_{N}\left(\omega_{\lambda }\right)}{P_{S}\left(\omega_{\lambda }\right)}=\frac{P_{rd}+\left|F\left[S_{m}\left(\lambda_{i}\right)\right]\right|}{{\left|F\left[S_{m}\left(\lambda_{i}\right)\right]\right|}^{2}}=\frac{P_{rd}+\left|G\left(\omega_{\lambda }\right)\right|}{{\left|G\left(\omega_{\lambda }\right)\right|}^{2}}.$$

When carrying out the averaging of the registered spectrum, the spectral power density of the signal *P_S_*(ω_λ_) changes slightly, and *P_N_*(ω_λ_) decreases *m* times, which must be taken into account when applying (10). Since the values of the instrumental function *h*(λ*_k_*) are normalized so that the area under it is equal to one, the ratio *P_N_*(ω_λ_)/*P_S_*(ω_λ_) must be multiplied by the square of the sum of the counts *h*(λ*_k_*), i.e., divided by Δλ^2^.

(11) $$H_{W}\left(\omega_{\lambda }\right)=\frac{H^{*}\left(\omega_{\lambda }\right)}{{\left|H\left(\omega_{\lambda }\right)\right|}^{2}+{\left(\sum_{k}h\left(\lambda_{k}\right)\right)}^{2}\frac{P_{N}\left(\omega_{\lambda }\right)}{m\cdot P_{S}\left(\omega_{\lambda }\right)}}=\frac{H^{*}\left(\omega_{\lambda }\right)}{{\left|H\left(\omega_{\lambda }\right)\right|}^{2}+\frac{1}{\Delta \lambda^{2}}\frac{P_{rd}+\left|G\left(\omega_{\lambda }\right)\right|}{m\cdot {\left|G\left(\omega_{\lambda }\right)\right|}^{2}}}.$$

In practice, it is convenient for the spectra correction algorithm to keep the area under the original and corrected spectral dependencies unchanged. For this, the transfer characteristic of the formed Wiener filter should be normalized by dividing it by a constant coefficient, which is *H_W_*(ω_λ_ = 0). Then, the first element of the normalized transfer characteristic *H_W__n_*(0) will always be equal to unity, which will ensure the keeping of the average value or the constant component of the corrected spectrum.

**Figure 8:**
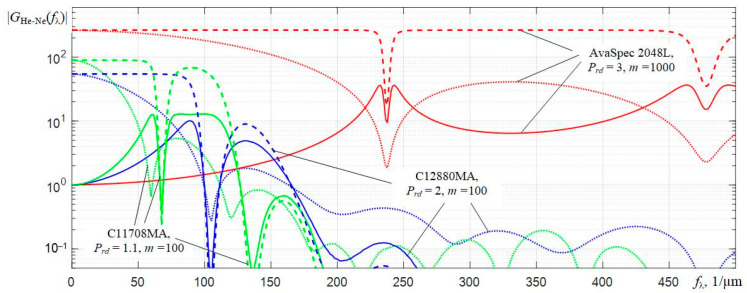
Moduli of the components of the Fourier transforms |*G*_He-Ne_(*f*_λ_)| of the original He-Ne laser spectra (dotted lines) obtained for three spectrometers, the moduli of the formed normalized transfer functions of the Wiener filter |*H_Wn_*_He-Ne_(*f*_λ_)| (solid lines) and the results of their multiplication (dashed lines) in the frequency range from 0 to 500 1/μm.

In order to understand the applied algorithm for increasing the resolution, the results of multiplying (dashed lines) of the initial He-Ne spectra *G*_He-Ne_(ω_λ_) (dotted lines), obtained by the three discussed spectrometers, by the corresponding dependencies *H_Wn_*_He-Ne_(ω_λ_) (solid lines) are depicted in Figure 8. They clearly show that filtering using *H_Wn_*_He-Ne_(ω_λ_) enhances the components of the Fourier transforms of the original He-Ne laser spectra in a certain frequency band. In this case, the corresponding constant shelves are formed, followed by a decrease in the *H_Wn_*_He-Ne_(ω_λ_)⋅*G*_He-Ne_(ω_λ_) dependencies in the higher frequency range of ω_λ_ values. The observed drop leads to a limitation of the frequency band of the product of the dependencies *H_Wn_*_He-Ne_(ω_λ_)⋅*G*_He-Ne_(ω_λ_).

**Figure 9:**
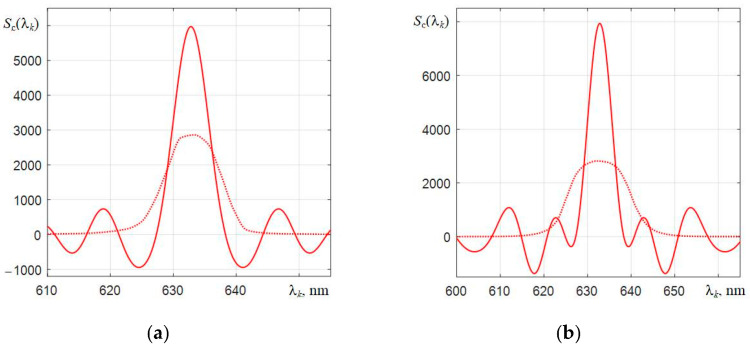
The original (dotted lines) and corrected by the Wiener filter (solid lines) spectra of the He-Ne laser, obtained with the spectrometers C12880MA (**a**) and C11708MA (**b**), respectively.

Therefore, when the inverse Fourier transform (3) is applied to this product, the shape of the corrected spectra $S_{r}^{*}\left(\lambda \right)$ is formed not as a δ-function (its Fourier transform has a uniform spectrum), but as a central response with the presence of lateral oscillations, which is illustrated by the dependencies in Figure 9. In a hypothetical case, i.e., in the absence of radiation noise and spectrometer noise, the corrected spectra of the He-Ne laser $S_{r}^{*}\left(\lambda \right)$ will have the form of a δ-function.

Since it is difficult to numerically compare the form of the product *H_Wn_*_He-Ne_(ω_λ_)⋅*G*_He-Ne_(ω_λ_) with the definition of the resolution *r* used by spectrometer manufacturers, it is possible to approximately estimate the corrected resolution *r_c_* at half maximum of the positive values of the central peak of the corrected spectra *S_c_*(λ*_k_*). These values were *r_c_* = 5.98 nm for C12880MA and *r_c_* = 5.93 nm for C11708MA.

**Figure 10:**
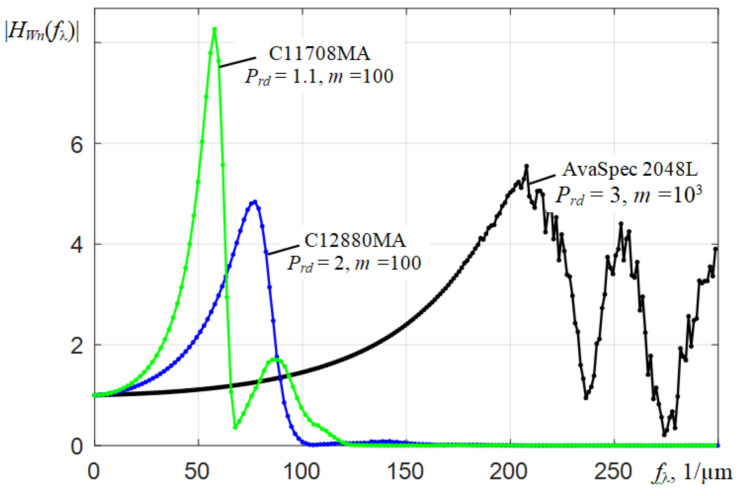
Transfer characteristics of the normalized Wiener filters |*H_W__n_*_LEDR_(*f*_λ_)| for three spectrometers using the Fourier transforms of the corresponding spectra of the red LED *G*_LEDR_(ω*_λ_*).

Fourier transforms of the spectra of LEDs *G*_LED_(ω_λ_) are falling when ω_λ_ dependencies rise. Therefore, the moduli of the normalized transfer functions of the Wiener filter |*H_Wn_*_LED_(ω_λ_)| fall much faster with ω_λ_ increasing in comparison with the dependencies |*H_Wn_*_He-Ne_(ω_λ_)| and contain no more than 2 clearly defined petals. The above is well illustrated by the dependencies obtained for the spectra of a red LED (Figure 10).

### 6.5. Analysis of the Efficiency of Applying Wiener Filtering in the Spectra Correction

Analysis of the dependencies shown in Figure 8 and Figure 10 shows that Wiener filtering in the case under consideration increasesseveral times the amplification of the components of the Fourier transforms *G*(*f*_λ_) of the interpolated spectra *S_m_*(λ*_k_*), which are located in the low-frequency region. Components in the high-frequency region, where the signal-to-noise ratio is much lower, are, on the contrary, attenuated. The efficiency of applying Wiener filtering can be assessed by comparing the corrected spectra of LEDs *S_c_*(λ*_k_*) with similar original spectra *S_m_*(λ*_k_*) registered by AvaSpec 2048L.

**Figure 11:**
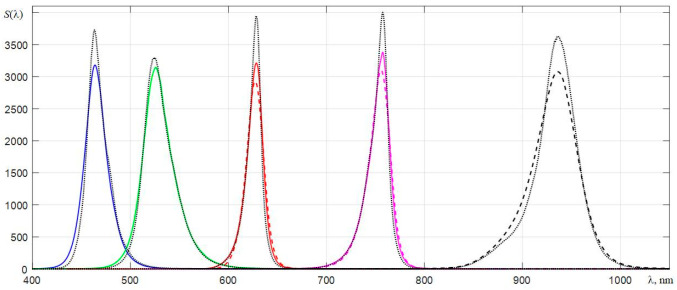
Normalized spectra of LEDs, which are registered using the AvaSpec 2048L (dotted lines), C12880MA (solid lines), and C11708MA (dashed lines) spectrometers.

In order to compare the spectra of LEDs, which were obtained with different spectrometers, their normalization was carried out, providing the same areas under them. Figure 11 shows the spectra of the five LEDs interpolated on a small scale of counts λ*_k_*, which were obtained by three spectrometers. The readout scales of the spectrometers are shifted to the right by the measured values Δλ*_ws_*, which are given in Section 6.2. The spectra of the four LEDs with blue, green, red, and dark red glow colors obtained by AvaSpec 2048L were normalized to the corresponding spectra obtained by C12880MA. The spectra of the two red and dark red LEDs obtained by C11708MA were normalized to the spectra registered by C12880MA. The spectrum of the infrared LED peaking at around 936 nm, obtained by AvaSpec, was normalized to the spectrum registered by C11708MA. It should be noted that the maxima of the normalized spectra of LEDs obtained by C11708MA (dashed lines) are slightly shifted to the left, which indicates a redistribution of the spectral components by the dispersing path of this spectrometer in such a way that the center of mass of the spectra of LEDs is shifted towards shorter wavelengths. This displacement is most probably formed by the shape of the concave diffraction grating used in this microspectrometer.

**Figure 12:**
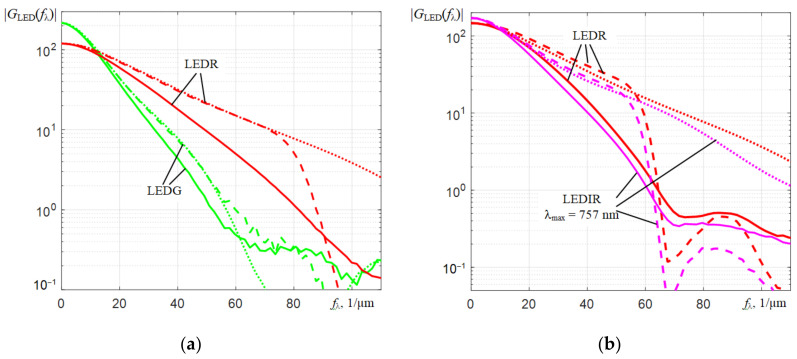
Moduli of the Fourier transforms of the original spectra (solid lines) and the corrected (dashed lines) of the LEDs registered by C12880MA (**a**) and C11708MA (**b**), and similar moduli of the Fourier transforms of normalized spectra obtained by AvaSpec 2048L (dotted lines).

**Figure 13:**
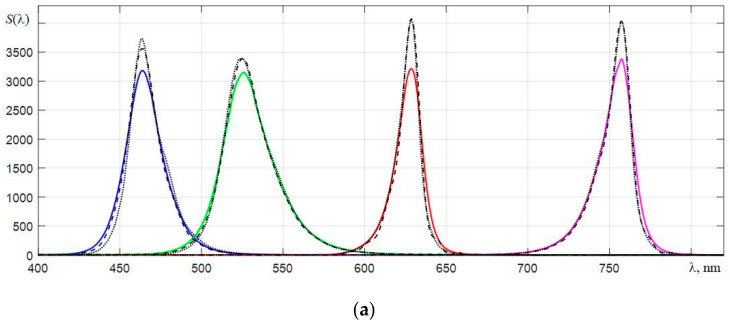

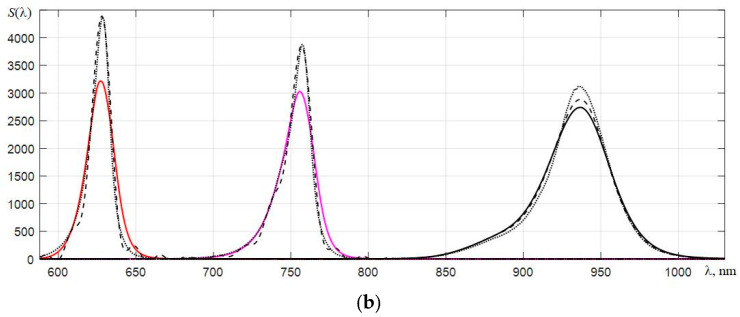
Spectra of LEDs *S_m_*(λ) registered by spectrometers C12880MA (**a**) and C11708MA (**b**) (solid lines), similarly normalized spectra *S_n_*(λ) obtained by AvaSpec 2048L (dotted lines), and the results of the correction of the spectra *S_m_*(λ) by Wiener filtering (dashed lines).

It is not worth expecting that after the Wiener filtering, the spectra obtained by different spectrometers will not exactly coincide, since they are affected by the spectral sensitivities of the spectrometers used, which differ from each other, especially at the wings of their spectral ranges. In addition, the form of the instrumental functions *h*(λ*_k_*) of microspectrometers can depend on the wavelength, i.e., their spectral resolution *r* can change especially near the boundaries of the spectral range. These differences can only be revealed by experimental measurements of the spectra with their subsequent correction. The considered filtering corrects the Fourier transforms *F*[*S_m_*(λ*_k_*)] of the initial spectra *S_m_*(λ*_k_*) of LEDs mainly in the region of the components of the first lobe of the Fourier transform of the instrumental function. As can be seen from Figure 12, the result of multiplying (dashed lines) the components of the Fourier transforms of the original LED spectra (solid lines) by the corresponding normalized transfer functions of the Wiener filter for LEDs with the parameters *P_rd_* and *m* indicated in Figure 10, approach well to the Fourier transforms of the spectra of these LEDs obtained by AvaSpec 2048L (dotted lines) in the region of low frequencies *f*_λ_. As follows from the graphs in Figure 13, a good agreement of the corrected spectra with the spectra registered by AvaSpec 2048L is observed for C12880MA. Only the corrected spectrum of the blue light-emitting diode has slight differences, which are due to the deviation of the instrumental function *h*(λ) of C12880MA in the short-wavelength part of the spectral range from *h*(λ) in the range from 500–800 nm.

For the C11708MA spectrometer, which has the worse spectral resolution *r* comparing with that for the C12880MA spectrometer, the corrected LED spectra contain noticeable fluctuations. Due to the suppression of a part of the spectral components in the region *f*_λ_ > 60 1/μm, the harmonic components are depleted, from which the corrected spectrum *S_c_*(λ*_k_*) is restored in the inverse Fourier transform. This leads to noticeable fluctuations in *S_c_*(λ*_k_*) due to their insufficient compensation by suppressed high-frequency components. This effect is also inherent in the corrected spectra obtained by C12880MA. However, due to the better initial spectral resolution *r* of C12880MA, these fluctuations are less pronounced. In the long-wavelength part of the spectral range of C11708MA, the spectrum correction is insufficient, which indicates a deviation of the instrumental function *h*(λ) from the dependence which is characteristic of it in the shorter wavelength region.

Approximate estimates of the equivalent spectral resolution of C12880MA and C11708MA (about 6 nm), which were obtained above in the analysis of the initial He-Ne laser spectra corrected by the Wiener filter (Figure 9), are doubtful. It is due to an incomprehensible influence of side oscillations on the shape of the central peak of the corrected spectra of the *S_c_*(λ*_k_*) of the He-Ne laser (solid lines in Figure 9). Therefore, we can propose another estimate of the corrected resolution *r_c_* based on the analysis of the behavior of the Fourier transform modulus *S_c_*(λ) corrected by Wiener filtering. By analogy with the terminology used in radio engineering, the estimate of the corrected resolution *r_c_* could be based on the cutoff frequency of the low-frequency filter. This is the frequency at which the amplitude of harmonic oscillations at the filter output decreases by 3 dB or by $\sqrt{2}$ times compared with that at the filter input. Analyzing the dependencies in Figure 12, it can be obtained that the similar cutoff frequency value *f*_λ*cf*_, at which the components of the Fourier transform corrected by the Wiener filter (dashed lines) decrease by $\sqrt{2}$ compared to the components of the Fourier transform of the spectral response (dotted lines) recorded by the AvaSpec reference spectrometer, are *f*_λ__CF_ ≈ 80 1/µm for C12880MA and *f*_λ__CF_ ≈ 60 1/µm for C11708MA. The period of these harmonic oscillations at the cutoff frequency *f*_λCF_ ≈ 80 1/µm is *T*_λ_ = 1000/80 = 12.5 nm for C12880MA and *T*_λ_ = 1000/60 ≈ 16.7 nm for C11708MA. One period of this harmonic oscillation can be represented as a spectral peak. The determination of *r_c_* at half-height of that peak (i.e., the determination of *r_c_* used by spectrometer manufacturers) can be applied to it. This approach gives more adequate estimates of the corrected (equivalent) resolution: *r_c_* ≈ 6.3 nm for C12880MA and *r_c_* ≈ 8.4 nm for C11708MA.

Comparing the spectral resolution of C12880MA and C11708MA before correction (10.2 nm and 15.2 nm, respectively) and after correction (equivalent resolution of about 6.3 nm and 8.4 nm, respectively), we can conclude that, in this case, noticeable fluctuations in the corrected spectra are absent if the equivalent resolution *r_c_* is improved by no more than 40%. Note that to reduce these oscillations, the original registered spectra *S_m_*(λ) should have a sufficient signal-to-noise ratio and also be smooth, i.e., their Fourier transforms must decrease with increasing *f*_λ_. In some cases of diffuse reflection spectroscopy with spatial resolution (at large distances ρ between the point of radiation entrance into a condensed scattering medium and the point of radiation registration), the recorded spectral dependencies can have a reduced signal-to-noise ratio. In such cases, the obtained spectral dependencies can be smoothed with a Savitzky–Golay digital filter. Its operation is based on the formation of a sliding window with a specified length and calculation of the averaged smoothed dependence by the least squares method. In this case, uncorrelated noise is suppressed well, and the distortions of the recorded spectral dependencies are minimal. Then, the Fourier transforms of the smoothed spectral dependencies will be sufficiently smooth, which will reduce the distortions of the corrected spectra due to the appearance of side oscillations.

To clarify in detail the possibility of reducing the level of oscillations in the corrected spectra registered by considered microspectrometers, additional studies are required, which should take into account the shape of the spectral sensitivity of their photodiode array and changes in the aperture NA when using focusing objectives, which requires their careful calibration with a reference emitter.

## 7. Conclusions

The conducted study of the possibility of using the C12880MA and C11708MA microspectrometers in inexpensive small-sized devices for optical diagnostics shows the need for a number of hardware and software solutions to improve their parameters and characteristics.

To reduce the cost of the device when connecting these spectrometers, it is advisable to use modern microprocessor-based debug boards, for example, those that are based on the STM32F103C8T6 microprocessor. Such boards have all the necessary electronic components for full interaction with microspectrometers and a control computer, the required volume of non-volatile flash memory and have small dimensions. When developing appropriate programs for processing the obtained spectra, it is necessary to apply algorithms that provide:

- Reduction of the influence of nonhomogeneity of dark counts of the spectrometer photodetector array;
- Correction of the registered spectra, eliminating the influence of the nonlinearity of the light characteristic of the spectrometer;
- The required offset of the reading scale λ;
- Transition to a linear scale of counts with a smaller sampling interval;
- Spline interpolation of the registered spectra on a uniform small scale of λ counts.

Due to the small size of the C12880MA and C11708MA microspectrometers, their spectral resolution *r* is limited. A slight improvement in the equivalent spectral resolution in the recorded spectra *S*(λ) can be provided by their subsequent additional digital processing. The analysis of the possibility of improving *r* by applying digital Wiener filtering to the Fourier transforms *G*(*f*_λ_) of the registered spectra *S_m_*(λ) and the subsequent implementation of the inverse fast Fourier transform shows the applicability of this approach with a limited frequency band of the Fourier transform *G*(*f*_λ_) and a sufficient signal-to-noise ratio, i.e., for sufficiently smooth initial spectra. Naturally, if it is necessary to apply correction *r*, then it is necessary to carry out operations to carefully measure the instrumental function of the spectrometer *h*(λ) in the used measurement circuit using laser radiation, the spectrum of which is close in shape to the δ-function.

Experimental testing of the considered method for improving the equivalent resolution *r* by digital Wiener filtering of the spectra of several LEDs shows that to ensure the smallness of fluctuation oscillations in the corrected spectra, it is necessary to ensure the smoothness of the initial spectra by averaging them. If the signal-to-noise ratio is insufficient, the smoothing of the interpolated spectra can be applied using the Savitzky–Golay digital filter, which, with a short sliding filtering window, weakly changes the shape of the registered spectrum and averages the high-frequency noise quite well. The real improvement in the equivalent resolution *r* for the corrected spectra subject to the condition of small side oscillations turns out to be small and amounts to about 40% relative to the spectral resolution, which is measured experimentally or declared by their manufacturer. For example, for the spectra of LEDs obtained with the C12880MA microspectrometer, the equivalent resolution determined at the level of 0.5 from the maximum of the instrumental function *h*(λ) can be improved from 10.2 to 6.3 nm. To reduce the level of these oscillations, it is necessary to carefully measure the instrumental function *h*(λ) taking into account the spectral sensitivity of the spectrometer, which is determined using the reference emitter.

Thus, the practical testing of the considered methods of correcting the spectra registered by the C12880MA and C11708MA microspectrometers shows that when the aforementioned algorithms are included in their software and the instrumental functions *h*(λ) are carefully measured, these spectrometers can be used in portable spectrometric equipment, designed to control the optical properties of condensed finely dispersed scattering media with smooth diffuse backscattering spectra.
